# Private Demand for Covid-19 Vaccine: A Contingent Assessment from a Low-and Middle-income Country

**DOI:** 10.22037/ijpr.2021.115008.15153

**Published:** 2021

**Authors:** Zahra Meshkani, Leila Zarei, Narges Hajimoladarvish, Jalal Arabloo, Aziz Rezapour, Hiro Farabi, Najmeh Moradi

**Affiliations:** a *Health Management and Economics Research Center, Health Management Research Institute, Iran University of Medical Sciences, Tehran, Iran. *; b *Health Policy Research Center, Institute of Health, Shiraz University of Medical Sciences, Shiraz, Fars, Iran. *; c *Department of Economics, Faculty of Social Sciences and Economics, Alzahra University, Tehran, Iran.*

**Keywords:** COVID-19, Contingent valuation method, Vaccine, Demand function, Willingness to pay

## Abstract

This study aimed to estimate Iranian willingness to pay (WTP) for a hypothetical COVID-19 vaccine and its determinants. A cross-sectional online survey was conducted from May 2^nd^ to 20^th^, 2020 among the general population of Iran to estimate WTP for hypothetical COVID-19 vaccines. Four scenarios with different levels of efficacy and duration of protection were presented to respondents in the payment card scale of the contingent valuation method (CVM). With the corresponding WTPs under diﬀerent scenarios, mean, trimmed mean, median WTP values, and vaccine demand was estimated. A semi-log regression model was employed to identify key factors. The vaccine acceptance rate and positive WTP were about 70% and 80%, respectively. The reluctant individuals believed free vaccination is a government responsibility. The highest trimmed mean and median WTP values were the US $15 and $4 for the vaccine with more than 80% efficacy and one-time vaccination. The median decreased to the US $2 in less effective scenarios. The vaccine demand was price-inelastic. Price, self-assessment virus risk, age, gender, education, income, and working in the health sector were significant factors. Given the price inelasticity of the COVID-19 vaccine, providing free vaccination by the Iranian government is highly recommended, particularly for low-income and vulnerable individuals.

## Introduction

COVID-19 is a virus with a high rate of person–to–person transmission and pathogenicity that appeared in December 2019 and endangered the lives of people around the world (1, 2). It is known as the most important crisis after World War II (3), leading to more than 1 million confirmed cases and 53,625 lost lives as of December 2020 in Iran (4). The outbreak imposes unprecedented financial pressure on health systems, particularly in the economics of hospitals to cure coronavirus patients who needed hospitalization, intensive care services, and ventilation (5). Treated patients need to undergo a recovery period at clinics with specific health care services, which, in turn, increased coronavirus patient treatment costs (6, 7). Because of the higher probability of hospitalization and mortality of COVID-19 as compared to other infectious diseases, it is shown that the medical costs of patients with COVID-19 are significantly higher (8).

The COVID-19 developed different clinical signs over time, making it more difficult to find treatment (8). While policymakers adopted preventive policies such as physical distancing, self-quarantine, and the use of protective products, the researchers are working on the vaccine (9, 10). The COVID-19 vaccines have reached the clinical stages, and recently three vaccines are approved (11).

Access to COVID-19 Tools (ACT) Accelerator is a collaboration between the world health organization (WHO) and scientists, businesses, and global health organizations to speed up the pandemic response, aiming to provide equitable access to COVID-19 diagnostics, treatments, and vaccines. COVAX is a pillar of ACT accelerator focused on universally providing safe and effective vaccination against COVID-19 (12) . However, policymakers need the information to design strategies for distributing and prioritizing the vaccine in the first stages of producing and distributing the vaccine as the world will face scarcity (10, 13). In the absence of actual transaction data, the stated demand in hypothetical situations can be estimated by the contingent valuation method (CVM) (14, 15). CVM is an economical technique used to assess individuals’ economic valuation of hypothetical goods through eliciting their willingness to pay (WTP) (16).

Many studies have used the WTP concept in health care, particularly for new vaccines, such as the hepatitis B vaccine in Pakistan in 2018 (17), the cholera vaccine in Bangladesh in 2008 (18), the Tibetan fever vaccine in Vietnam in 2006 (19), AIDS vaccine in Thailand in 2006 (20) . Recently WTP is used for assessment of COVID-19 vaccine in Chile (13), Ecuador (21), Indonesia (2), Romanian (1), and China (22).

Although the COVID-19 vaccines, Pfizer-BioNTech, Moderna, and AstraZeneca/Oxford, have been approved and the vaccination is now implementing in US, UK, and some other countries and some vaccines are in the human clinical phase, providing evidence on acceptance. Monetary value of vaccine could help policymakers to choose the best strategy (23).

Thus, this study aimed to estimate Iranian demand for a hypothetical COVID-19 vaccine to help policymakers to assess public willingness to vaccinate, public preference to take the vaccine by their income or government budget, the monetary value of vaccine, and finally estimating vaccine demand, it’s affecting factors and net private benefit value of vaccination. 

## Experimental

*Study design and setting*

This study used a cross-sectional online survey that was distributed between May 2^nd^ to 20^th^, 2020, across Iran. Different hypothetical COVID-19 vaccine scenarios were designed using the state-preference WTP method to estimate private vaccine acceptance and demand. 

*Study population, sampling and sample size*

The study population were all Iranians aged more than 18 years old who had access to the internet in all provinces of Iran. We used a mixed sampling process (convenience and snowball sampling). In this way, at the beginning of the survey, a focal point was selected to distribute the questionnaire link in each province. There was not a sampling framework and responses to the questionnaire were completely random. To this end, the link was sent to anyone who could only answer anonymously and/or send it to anyone else; hence, the questionnaire link was sent randomly and distributed in the form of snowballs by the focal points and previous participants. According to Harapan *et al.* (23) study, the sampling adequacy was determined. In addition, an invitation letter and a written consent form that included information about research purposes and ethical issues were provided for the individuals.

*Study instrument*

The study instrument was a structured questionnaire including three main parts: COVID-19 virus risk assessment, respondents’ characteristics, and WTP for hypothetical COVID-19 vaccines. Before asking questions, updated information about the number of COVID-19 cases, deaths, and recovered ones in Iran was presented to participants with a website link of “world meters coronavirus info” (24). 

Here are the details of each section:

*A) Risk assessment*

Three questions were designed to assess the risk of coronavirus on a 5-point Likert scale as follows:

How dangerous do you consider *corona-virus* to be?

How much do you consider yourself to be exposed to the coronavirus?

How much do you think your family members are at risk for coronavirus?

*B) Health and social-demographic char-acteristics*

Age, educational level, gender, job monthly income level, family size, the number of family members over 60-year-old as well as under 10-year-old, the existence of members with chronic disease, expected life expectancy, insurance coverage, province of residence, exposure to Covid-19 by themselves or family members were included as health and social-demographic variables.

*C) WTP for COVID-19 vaccines*

**Figure 1:**
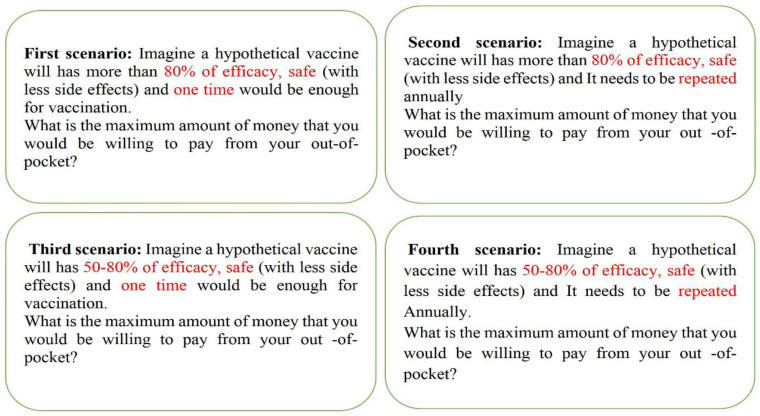
Hypothetical scenarios for COVID-19 vaccine

Four scenarios were defined adapted from previous studies (1, 2, 13, 21, 23, and 25-27). The respondents explained efficacy, time of vaccination, and possible side effects in these scenarios. The participants were asked about their maximum willingness to pay in each scenario. Figure 1 shows the hypothetical scenarios.

The respondents indicate their WTP on a payment card scale ranging from 500,000 IRR (= US$2.71) to more than 200,000,000 IRR (=US$ 870) per vaccine. Moreover, respondent could state their WTP if it was not on the scales. A pilot study with 60 individuals was conducted to determine these values through a WTP open-ended question. 

Due to high fluctuation in the exchange rate, to convert values to US dollar, we used a simple moving average during the previous 200 days. Accordingly, US $1 is equal to 230,000 IRR.

*Data analysis*

Descriptive statistics were presented as frequency, percentage, and mean for the socio-economic demographic variables. WTP for all of the scenarios was presented as mean ± standard deviation, median, non-zero minimum, and maximum. 

To predict the Covid-19 vaccine demand function in each scenario, the respondents’ WTP for the hypothetical vaccine and the number of vaccines that would be purchased at that price were used as Price and Quantity, respectively. 

To model a Covid-19 vaccine demand, a semi-log regression model was used. Key variables were socio-economic demographics and Coronavirus risk assessment. 

Excel 2016 and R software were used for data management and analysis, respectively. 

## Results

*Participants’ characteristics*

Table 1 shows the health and socio-economic demographics and health characteristics of respondents. A total of 2524 people viewed the online questionnaire, and 878 people take part in the study. -Third of respondents resided in Tehran -the capital of Iran; the remaining were from other provinces. Seventy-eight percent of participants were female. The majority of respondents (75.5%) were in the 18-39 age group. About half of the respondents have a university degree. In total, 48% have a job, and 18% of them worked in health sector. Seventeen percent stated that they have no income, and 70% indicated that their monthly income is less than IRR 50.000.000 (US $217). Most of the participants (85%) had health insurance.

They were having chronic diseases such as cardiovascular and diabetes was seen in respondents and their family member as 11% and 51%. About 6% of the sample stated that at least one of themselves or their family members including themselves had infected by a coronavirus- It should be mentioned that the reason of low infected rate is that this study was performed in the first peak of the Covid-19 outbreak. 

*COVID-19 Risk assessment and vaccine acceptance*

**Figure 2:**
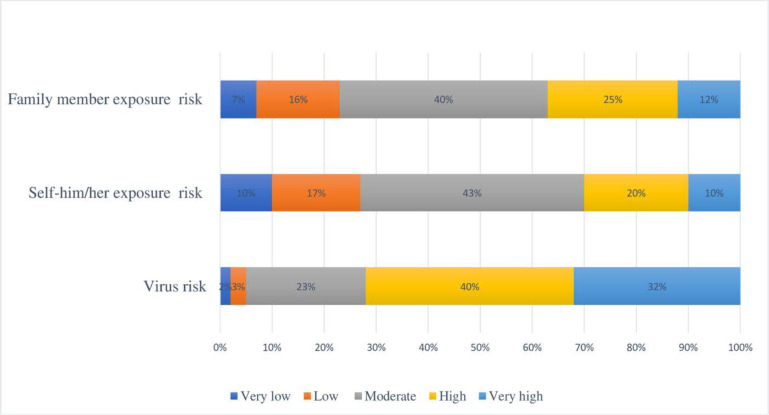
The COVID-19 risk assessment

While about 73 percent of the participants considered the risk of the disease as high or very high, about 40% considered a moderate risk of exposure for themselves and their family members. See Figure 2 for details. 

**Figure 3:**
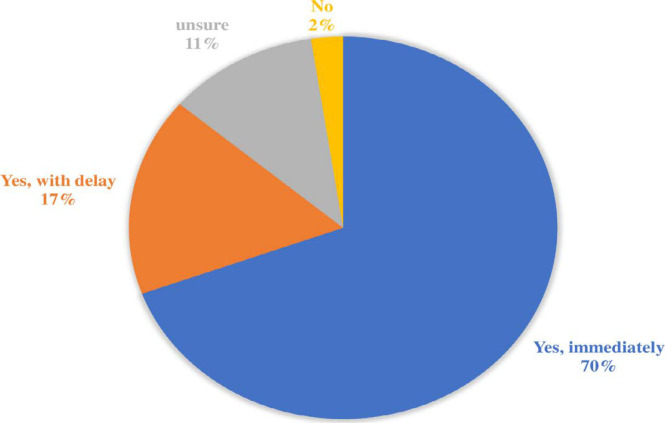
Risk assessment of COVID-19 virus

For the vaccine acceptance (Figure 3), 2% of respondents were reluctant, and 11% were unsure. Most respondents declare that they would take it immediately (70%) or get a vaccine with some delay (17%). 

*WTP for the COVID-19 vaccine*

Generally, 80% of participants had positive WTP for a hypothetical coronavirus vaccine. The reluctant respondents believed that it is a government responsibility to provide free vaccination. Table 2 shows WTP for the vaccine in different scenarios. The highest value was USD $22 for a vaccine with 80% efficacy and one-time vaccination. The WTP is decreasing due to a reduction in efficacy and duration protection. Compared to the first scenario, the mean WTP is decreased by 30% in the fourth scenario. To eliminate the influence of outliers, the 5% trimmed mean (5%) were 15,12,11,10 for scenarios 1 to 4, respectively. The median WTP in all scenario-except the first scenario- is about $2. 

*Covid-19 vaccine demand and critical factors*

**Figure 4:**
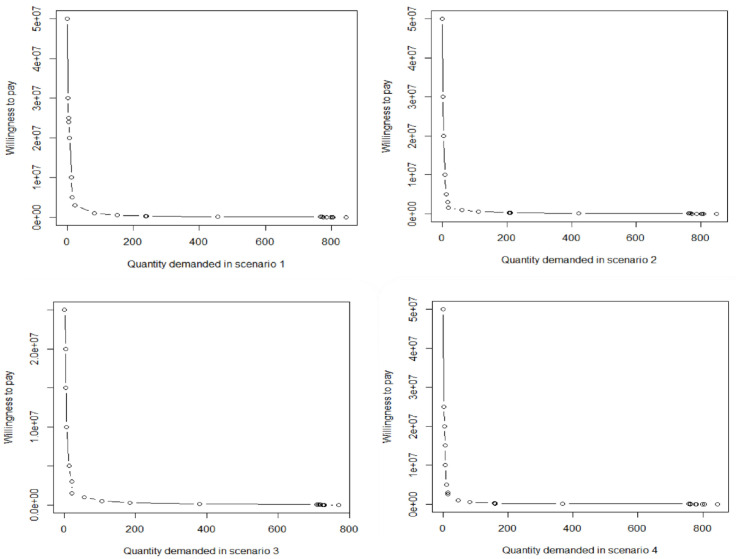
COVID-19 vaccine demands

Figures 4 show the vaccine demands each scenario across Iran. The quantity demanded at any given price is calculated as the total number of vaccines purchased. As it is revealed, all demand curves had a hyperbolic shape. The number of individuals who can aﬀord the vaccine at IRR 500000 ($2) price decreases from 766 to 7 for IRR 200000000 ($870) or higher. Totally, in all scenarios, about 50% of respondents had WTP about $2.

We estimate a linear model of COVID-19 vaccine demand to investigate how price and other covariates influence demand for the COVID-19 vaccine. Given the high number of categorical explanatory variables, we log transformed quantity demanded to reduce its variance and balance the dependent variable’s scale with most independent variables. Table 3 shows the variables. 

Table 4 presents parameter estimates of demand for vaccine under 4 diﬀerent scenarios with varying efficacy and protection duration. In all 4 models the coeﬃcient of price is negative and signiﬁcant at 1%. More precisely, for every IRR1000,000 ($4.3) increase in price of vaccine, the quantity demanded decreases by 2.075 to 3.325 percent.

Here we focus below on the estimates from the ﬁrst model. Apart from price, the demand for vaccines decreases with the age and education of respondents. Ceteris paribus, older participants may underestimate their risk of contracting. Moreover, individuals with higher education may be more cautious about the vaccine that has not been tested yet. Since the coeﬃcient of gender is negative, ceteris paribus, as compared to females, males’ willingness to pay for the vaccine is lower. This is consistent with general ﬁndings of more risk-taking behaviors of males. Furthermore, individuals who are working in the health sector have higher demand for the vaccine. The exposure of people working in the health sector to consequences of COVID-19 may have driven this result. Ceteris paribus, the number of vaccines demanded increases with family size. In addition, people with no income have higher demand for the vaccine. This can be due to two reasons. First, it is optimal for those with no income to pay for vaccine instead of potential out of pocket cost of hospitalization. Second, individuals with no income are youngsters who do not work but can aﬀord the vaccine with family support. Since mean and median WTP values for individuals with no income are higher than the whole sample, our evidence supports the first explanation. Last, given the more concentration of wealth in capital (Tehran), other things equal, those who live in Tehran have higher WTP for the vaccine.

*The private surplus*

As mentioned earlier, recently three COVID-19 vaccines have been approved and the vaccination is now being implemented in the US, UK, and some other countries. They are Pfizer-BioNTech, Moderna, and AstraZeneca/Oxford with approximate prices of $20, $32-37, and $3-4, respectively (28).

 In order to compute individuals› private benefits of available vaccines in the market, we compute consumer surplus as a difference between individuals› WTP and the price of the vaccine. Although these vaccines could be purchased by governments at a lower price through negotiations, in the current study, we used the above-mentioned prices for calculations. Summing overall individuals, the average benefits from the cheapest available vaccine ($3) is only $20 as for 46% of our sample. Their WTP is less than $3. Also, considering the most expensive available vaccine ($37), there is no overall private surplus in our sample. Since there are a few individuals with extremely high WTP for the vaccine, it is better to consider the median consumer surplus which is $1.34. 

## Discussion

To our knowledge, this study was the first attempt to assess private demand for the COVID-19 vaccine in Iran. Our findings show that while vaccination is essential to the Iranian people, with more than 70% willingness to vaccinate and 80% cheerful willingness to pay, about 10% were protest respondents considering the provision of free vaccination as a government responsibility. 

The highest trimmed mean of WTP was about the US $15 (Int$ 152.6). The WTP decreased to the US $12 (Int$ 122.9), US $11 (Int$108.9), and the US $10 (Int$101.1) for vaccines with less efficacy or duration of protection. Generally, 50% of the sample have a WTP of less than the US $2.2 (Int$ 23.2). Just 0.07% of respondents were willing to pay more than the US $588 (Int$ 5942). 

According to García and Cerda, the mean WTP of the Chilean population for the COVID-19 vaccine was estimated at the US $184.72 (Int$ 334.8), and the acceptance rate was about 90% (13). In spite of the high vaccine acceptance rate in Chile and Iran, the results on the mean value of WTP for the vaccine were in contrast. This might be due to differences in income as, unlike Chile, Iran is not a high-income country. Moreover, high inflation across 2020 and before has led to a substantive decrease in real income and low purchasing power for Iranians. 

Sarasty et al analyzed the demand for the COVID-19 vaccine by a WTP method in Ecuador (21). They presented a hypothetical vaccine with two levels of efficacy (70% and 98%) and the protection duration (1 and 20 years) for a hypothetical vaccine in their online survey. The results showed that the acceptance rate of vaccination and WTP for the vaccine was about 97% and 85% of the respondent, respectively. Sarasty revealed that the protection duration was the key determinant of vaccine WTP. Other key factors were residence region, income, perceived hospitalization, and employment status. The estimated mean values are between Int$ 283.5 to Int$ 377.8, while the median WTP values ranged from the US $76.9 to the US $102.5 (Int$ 147.7 to Int$ 196.9) (21). Despite the high risk of the COVID-19 virus, the acceptance rate among Iranians was lower than Sarasty study despite similarity in the rate of positive WTP Moreover, our findings showed association of WTP to gender, age, education, region of residence, income, being a health worker, self-risk, and family size.

Berghea *et al.* used the Van Westendorp Price Sensitivity Meter method to estimate the WTP of Romanians. In their study, the acceptable price range for a hypothetical COVID-19 vaccine was between €20 and €200 (Int$ 12.2 and Int$ 121.8) (1). Their finding revealed that WTP was similar in all subgroups. However, wealthy individuals selected a €50 to €400 (Int$ 30.4 to Int$ 243.6) interval. 

Harapan *et al*., investigate the acceptance rate of a COVID-19 vaccine in Indonesia. They presented a vaccine free of charge with 95% or 50% efficacy to participants. About 93% of the respondent would like to be vaccinated by a vaccine with 95% efficacy. In the Harapan study, working in health care and a higher perceived risk of the virus were the associated factors for acceptance of the vaccine (2). In another study, using a simple dichotomous contingent valuation approach, Harapan examined WTP for a COVID-19 vaccine and its associated determinants in Indonesia. Their results show that 1,065 (78.3%) respondents were willing to pay for the COVID-19 vaccine with a mean and median WTP of US $57.20 and the US $30.94 (Int$175 and Int$ 97.4), respectively. High perceived risk, working in the health sector, and having a high income were associated with higher WTP. These finding is in line with the present study which those with health care job or higher self-exposure virus risk have higher demand for vaccine (23).

Dodd *et al.* (2020) investigated the acceptance rate of the COVID-19 vaccine through an online survey with 4362 adult Australians in April. Their results showed that while the majority of 85.8% (3741) will vaccinate if it became available, 4·9% (213) said they would not get the vaccine, and 9·4% (408) were indifferent (27). In our study, only 2% were unwilling to vaccinate, and 11% were unsure. 

Armand *et al. *(2020) surveyed the Indian population to investigate the willingness to vaccinate in the COVID-19 crisis (29). They revealed that, contrary to advanced economies, stated willingness to vaccinate is high among this slum dweller population. Of those surveyed, 95% would like to get the vaccine for COVID-19, while willingness in seven European countries (UK, Netherlands, Portugal, Italy, Germany, France, and Denmark) was at only 74% in April 2020, despite most of these countries experiencing their first peak of cases at this time. Importantly though, in addition to 5% of the population not being willing to get vaccinated, 36% stated that they would only get vaccinated if they did not have to pay for it (29). This rate is near to Iranian acceptance rate.

Generally, the current literature revealed that the COVID-19 vaccine acceptance rate is relatively high in the globe. Moreover, there is high positive WTP rate for vaccine. However, there is a considerable difference in mean and median WTP for vaccine between countries. For instance, higher income countries with lower budgetary constraints revealed higher value for such as Chile in compared Iran. This may be because of high inflation and negative impact of Corona virus on economics situation of Iranian households. Moreover, estimating private surplus shows that there in on overall surplus at vaccines with high price. No benefit value could affect society participation in vaccination program. So, to maximize social benefits of vaccination and in response to high willingness to vaccinate, the recommend strategy are free vaccination by government, particularly for vulnerable individuals.

Finally, the results of this study are time-dependent because not only being in different phases of the epidemic (in terms of peak or severity of infection and death) but also the effect of media and positive and negative advertising about the vaccine can affect the perception of participants and their vaccine monetary valuation. However, what justifies doing such research is identifying influencing factors and providing information to decision-makers to improve evidence-based decision-making.

**Table 1:** Characteristics of participants

	**Characteristic**	**Frequency (%)**
	Total	878 (100%)
Gender	Female	676 (77.7%)
	Male	195 (22.2%)
	Missing	7(1%)
Age group	18-28	287 (32.7%)
	29-39	368 (41.9%)
	40-50	172 (19.6%)
	51-60	38 (4.3 %)
	> 60	7 (0.8%)
	Missing	15 (1.7%)
Geographical location	Capital (Tehran)	323 (36.8%)
	Other province	549 (62.5%)
	Missing	6 (0.68%)
Education	Elementary	34 (3.9%)
	Diploma	223 (25.4%)
	University	613 (70%)
	Missing	8(1%)
Income level	Less than US $118	201 (22.9%)
	Between US $118- 294	318 (36.3%)
	Between US $ 294-588	119 (13.6%)
	More than US $ 588	29 (3.3%)
	No income	145 (17%)
	Missing	66(8%)
Family size	1 to 2	186 (21.2%)
	3 to 5	629 (71.6%)
	>6	57 (6.5%)
Health Insurance	Basic insurance	747 (85.08%)
	no insurance	123(14.01%)
	Missing	8 (0.91%)
Chronic Disease history	Themselves	49(6.2%)
	Family member	362 (46%)
	both (Themselves and Family member)	48(6.1%)
	None	419 (53.2%)
Infected by corona virus	Themselves	18 (2.1%)
	Family member	37 (4.2%)
	No-one	823 (93.7%)

**Table 2:** The WTP values (US $) for the hypothetical COVID-19 vaccine in Iran

**WTP values**				
	**Scenario 1 (more than 80% efficacy, one time)**	**Scenario 2 (more than 80% efficacy, repeated annually)**	**Scenario3 (50-80% efficacy, one time)**	**Scenario4 (50-80% efficacy, repeated annually)**
**Mean ± SD**				
IRR	5743919 ± 24656957	4914018 ± 24167710	4475366 ± 22207096	4020537 ± 21964285
US$	25 ± 107	21 ± 100	19 ± 97	17 ± 95
**Trimmed mean (5%)**				
IRR	3,471,928	2,797,378	2,480,620	2,301,930
US$	15	12	11	10
**Median**				
IRR	1,000,000	500,000	500,000	500,000
US$	4	2	2	2
**Mode**				
IRR	500,000	500,000	500,000	500,000
US$	2	2	2	2
**Non-zero minimum**				
IRR	100,000	100,000	100,000	100,000
US$	0.4	0.4	0.4	0.4
**Maximum**				
IRR	500,000,000	500,000,000	500,000,000	500,000,000
US$	2173	2173	2173	2173

**Table 3:** Description of variables

**Name**	**Type**	**Values**	**Explanations**
Price	Continuous	[0, 20000000]	
Gender	Categorical	Female = 1, Male = 2	
Age	Continuous	[18, 94]	
Education	Categorical	1, 2, 3, 4	It is in ascending order from Primary school to higher education (PhD)
City	Categorical	Others = 1, Tehran = 2	
Income	Categorical	1, 2, 3, 4, 5	It is in ascending order, except 5 refers to no income
Health Job	Categorical	1, 2	It is equal to 1 if respondent is working in the health sector
Family size	Continuous		
COVID-19 virus risk	Continuous	[1, 5]	It is in ascending order and shows respondents perception of Covid-19 risk
COVID-19 self-risk	Continuous	[1, 7]	It is in ascending order and shows respondents perception of Covid-19 infection risk to him/hers self
COVID-19 family risk	Continuous	[1, 8]	It is in ascending order and shows respondents perception of Covid-19 infection risk to their family member
Insurance	Categorical	1, 2, 3	It is in ascending order

**Table 4:** Estimated demand for COVID-19 vaccine

**Dependent variable**	**Log (Q)**			
	**Scenario 1**	**Scenario 2**	**Scenario 3**	**Scenario 4**
Price	−2.076e-08^∗∗∗^	−2.185e-08^∗∗∗^	−3.325e-08^∗∗∗^	−2.168e-08∗∗∗
	(0.000)	(0.000)	(0.000)	(0.000)
Gender (Male)	−0.184^∗∗∗^	−0.169^∗∗^	−0.184^∗∗^	−0.182^∗∗^
	)0.070(	)0.075(	)0.074(	)0.0780(
Age	−0.002	-0.004	−0.003	−0.005
	)0.003(	)0.003(	)0.003(	)0.003(
Education (College degree)	−0.084	−0.077	−0.072	−0.136
	)0.130(	)0.139(	)0.144(	)0.144(
Education (Bachelor and Master degree )	−0.283^∗∗^	−0.302^∗∗^	−0.297^∗∗^	−0.365^∗∗∗^
	)0.126(	)0.135(	)0.140(	)0.141(
Education (Ph.D. degree)	−0.155	−0.212	−0.191	−0.297∗
	)0.143(	)0.153(	)0.157(	)0.159(
City (Tehran)	0.377^∗∗∗^	0.381^∗∗∗^	0.345^∗∗∗^	0.388∗∗∗
	)0.050(	)0.053(	)0.053(	)0.055(
Income (Less than US $118)	0.088	0.107	0.087	0.064
	)0.069(	)0.074(	)0.075(	)0.077(
Income (Between US $118- 294)	−0.019	−0.029	−0.086	−0.031
	)0.069(	)0.074(	)0.074(	)0.077(
Income (Between US $ 294-588)	0.144	0.173^∗^	0.125	0.192^∗^
	)0.090(	)0.097(	)0.095(	)0.101(
More than US $ 588)	0.274^∗^	0.305^∗^	0.353^∗∗^	0.294^∗^
	)0.146(	)0.157(	)0.164(	)0.163(
Working in the health sector	−0.276^∗∗∗^	−0.308^∗∗∗^	−0.326^∗∗∗^	−0.356^∗∗∗^
	)0.069(	)0.073(	)0.073(	)0.076(
Family Head	−0.068	−0.045	−0.041	−0.099
	)0.075(	)0.081(	)0.081(	)0.085(
Family Size	0.035^∗∗^	0.034^∗^	0.011	0.029
	)0.017(	)0.019(	)0.018(	)0.019(
The number of family members aged less than 10-years old	0.047	0.042	0.029	0.063^∗^
	)0.030(	)0.032(	)0.033(	)0.033(
The number of family members aged less than 10-years old	−0.026	−0.021	−0.022	−0.016
	)0.026(	)0.028(	)0.027(	)0.029(
Basic insurance	0.032	0.071	0.135^∗^	0.055
	)0.073(	)0.078(	)0.078(	)0.081(
Complementary Insurance	−0.007	0.020	0.084	−0.000
	)0.083(	)0.089(	)0.088(	)0.092(
Expected life expectancy	−0.002	−0.001	−0.001	−0.002
	)0.001(	)0.001(	)0.001(	)0.002(
Covid-19 virus risk	0.004	0.004	−0.021	0.011
	)0.028(	)0.030(	)0.030(	)0.032(
Covid-19 self-risk	0.052^∗∗^	0.053^∗^	0.053^∗^	0.044
	)0.026(	)0.028(	)0.027(	)0.029(
Covid-19 family-risk	0.016	0.022	0.002	0.012
	)0.025(	)0.026(	)0.026(	)0.028(
Constant	6.004^∗∗∗^	5.964^∗∗∗^	6.069^∗∗∗^	6.234∗∗∗
	)0.256(	)0.273(	)0.272(	)0.284(
Observations (Valid)	842	846	768	843
R^2^	0.492	0.427	0.498	0.399
Adjusted R^2^	0.478	0.411	0.484	0.382
Residual Std. Error	0.669 (df = 819)	0.717 (df = 823)	0.680 (df = 745)	0.745 (df = 820)
F Statistic	35.989^∗∗∗^	27.837∗∗∗	33.658∗∗∗	24.705∗∗∗
	(df = 22; 819)	(df = 22; 823)	(df = 22; 745)	(df = 22; 820)

Note: ^∗^*p* < 0.1; ^∗∗^*p* < 0.05; ^∗∗∗^*p* < 0.01.

The number of years that a person is expected to live.

## Conclusion

Like other people in the world, the Iranian people would like to be vaccinated against the COVID-19. However, compared to other countries, Iranians willingness to pay for a Covid-19 vaccine is very low. This may be because of high inflation and the negative impact of Coronavirus on the economic situation. Considering high positive externality of the Covid-19 vaccine, it is recommended that the government finance it entirely. In the case of government budgetary constraints, allocating a high subsidy for the low-income and vulnerable individuals and providing vaccine privately by high-income households are recommended strategies. 

## Author contributions

 NM, ZM contributed to the conception and design of the study. ZM and JA, and HF collected the data. NH and NM analyzed the data. LZ, ZM, and JA, and AR drafted the first manuscript. NM, LZ, and NH revised the manuscript substantially. All authors read and approved the final manuscript.

## Funding

 This study was funded by Iran University of Medical Sciences (IUMS): Grant No: 99-1-48-17688, Ethical code: IR.IUMS.REC.1399.078).
